# Somatostatin Receptor Imaging in the Diagnosis and Management of Parathyroid Neuroendocrine Neoplasia

**DOI:** 10.3390/diagnostics14232718

**Published:** 2024-12-03

**Authors:** Uliana Tsoy, Karina Pogosian, Daria Ryzhkova, Olga Yudina, Ksenia Yakovenko, Pavel Ryazanov, Irina Matsueva, Polina Sokolnikova, Maksim Salov, Tatiana Karonova, Elena Grineva

**Affiliations:** Almazov National Medical Research Centre, 2 Akkuratova Street, Saint Petersburg 197341, Russia; utsoi@mail.ru (U.T.);

**Keywords:** parathyroid neuroendocrine neoplasms, parathyroid adenoma, somatostatin receptors type 2, somatostatin receptors type 5, PET/CT with 68Ga-DOTA-peptides

## Abstract

Background: Parathyroid tumors are classified as parathyroid neuroendocrine neoplasia (NEN) by the IARC-WHO classification. These tumors can occur with NENs from other sites, which often require total-body [68Ga]-DOTA-peptides PET/CT. This study aimed to assess the utility of [68Ga]-DOTA-peptide PET/CT in imaging parathyroid NENs and to evaluate the underlying mechanisms. Methods: Fifty patients with primary hyperparathyroidism (PHPT) and parathyroid NENs histologically confirmed as parathyroid adenomas (PAs) were included. PET/CT with [68Ga]-DOTA-peptide was performed in 16 patients with localized PAs, including 10 with MEN1 syndrome. Somatostatin receptor types 2 and 5 (SST2 and SST5) staining was performed on PAs from 48 patients. Somatostatin analogs (SSA) were prescribed in four patients with MEN 1 syndrome and 1 with persistent acromegaly, all with PAs and PHPT. The therapy effects on calcium and parathyroid hormone (iPTH) were evaluated. Results: [68Ga]-DOTA-peptide PET/CT detected 20 PAs with high radiopharmaceutical uptake. SST2 expression was negative on PA cell membranes in all cases and positive on endothelium in 39 (81%) PAs. Membrane SST5 expression was positive in 25 (52%) PAs and endothelial was positive in 40 (83%). Serum calcium levels decreased in patients on SSA therapy, while iPTH did not. Conclusions: PET/CT with [68Ga]-DOTA-peptides can detect parathyroid NENs. The incidental detection of high [68Ga]-DOTA-peptide uptake in the parathyroid region during whole-body PET/CT may prompt biochemical evaluation for PHPT. We suggest that endothelial SST expression mediates high radiopharmaceutical uptake by PAs and that SSA treatment can reduce hypercalcemia in PHPT patients.

## 1. Introduction

According to the IARC-WHO classification updates, parathyroid tumors are classified as well-differentiated neuroendocrine neoplasia (NEN): low grade—parathyroid adenomas (PA), and intermediate/high grade—parathyroid carcinomas [1]. Most parathyroid neuroendocrine neoplasms (PNENs) present as PA, the main cause of primary hyperparathyroidism (PHPT). In 85–90% of cases, PAs are single, and in 10–15% of cases, they are multiple. Carcinomas are the rarest parathyroid NENs, they occur in 0.5–1% of cases [2,3,4]. In the global population, the frequency of PHPT is 0.84%, and it is ranked third by prevalence amongst endocrine disorders, following diabetes mellitus and thyroid diseases [5]. PHPT patients suffer from an increased fracture risk, higher cardiovascular morbidity, and overall mortality compared with the general population [6]. Parathyroidectomy (PTX) is the only way to cure the disease [2,7]. Most of the PNENs are sporadic, but in some cases, they may be genetically determined. Multiple neuroendocrine neoplasia syndromes (MEN 1, MEN 2A, and MEN 4), are the main genetic pathologies associated with PNENs; they may be familial or occur due to de novo mutations [8,9,10,11,12]. All these syndromes are characterized by NENs of various localizations: gastro-entero-pancreatic, pituitary, and others in MEN1 and MEN4 syndromes; medullary thyroid carcinoma and pheochromocytoma in MEN2A [8,9,10,11,12]. Furthermore, considering the rather high prevalence of PHPT in the population, the co-existence of sporadic PNENs with NENs of other localizations is rather likely, especially in elderly people. The course of sporadic or genetically determined PHPT is indolent in many cases, and quite often the initial patient evaluation is driven by suspicion of an NEN in a location other than the parathyroid glands.

According to current views, [68Ga]-DOTA-peptides positron emission tomography/computed tomography (PET/CT) represents the gold standard in functional imaging for the assessment of well-differentiated NENs in conjunction with anatomic imaging (CT or MRI) [13,14]. The diagnostic value of this method is especially high in cases of multiple NENs. Its major advantage is the ability to detect tumors with different localizations, particularly when CT in PET/CT is performed with contrast enhancement. During these examinations, a focus of high [68Ga]-DOTA-peptides uptake in parathyroid glands’ allocation may be detected [15]. In such cases, questions arise if these findings may initiate biochemical evaluation for PHPT and if confirmed, is additional PNEN imaging necessary? First-line methods of parathyroid visualization include neck ultrasound combined with single-photon emission computed tomography/computed tomography (SPECT/CT) or scintigraphy with [99mTc]-sestamibi (MIBI) [16,17,18]. PET/CT with [18F]-choline may be used as a potential “alternative” first-line method whenever possible [19,20]. In some cases, when the standard imaging fails, additional diagnostic procedures may be considered. There are several second-line techniques available after negative or inconclusive first-line imaging, such as PET/CT with [11C]-methionine or [11C]-choline, and four-dimensional computed tomography (4D-CT) [17,18,19,20]. Overall, the currently used techniques for parathyroid visualization show high levels of specificity and sensitivity [17,18]. In some cases, particularly when several parathyroid glands are affected, the search for PAs is difficult and time-consuming, which results in a PTX delay and in an increased risk of PHPT-associated complications [6].

Thus, confirmation of [68Ga]-DOTA-peptides PET/CT capabilities to localize PNENs eliminates the need to perform additional long-term, complex, and expensive examinations and reduces the time before the surgery. The ability of MEN syndrome associated PAs to accumulate [68Ga]-DOTA-TATE was demonstrated previously [15]. However, it is unknown whether [68Ga]-DOTA-peptide accumulation is unique to MEN-associated PNENs or a common characteristic of all PNENs. The factors mediating [68Ga]-DOTA-peptides uptake in PNENs are also not disclosed yet.

The aim of our study was to investigate if MEN1-associated and sporadic PNENs may be detected using whole-body PET/CT with [68Ga]-DOTA-peptide to elucidate the mechanisms of [68Ga]-DOTA-peptide uptake in PNENs, and to clarify the potential of first-generation somatostatin analog (SSA) therapy for PHPT treatment.

We found that not only MEN1-associated but also sporadic PNENs may be detected using PET/CT with [68Ga]-DOTA-peptides. Therefore, the incidental detection of high [68Ga]-DOTA-peptide uptake in the parathyroid region during the whole-body PET/CT may initiate biochemical evaluation for PHPT. We assume that high radiopharmaceutical uptake by PAs is mediated by somatostatin receptor (SST) expression on endothelial cells. SSA treatment may mitigate hypercalcemia in PHPT patients, but this effect is not mediated by a decrease in the parathyroid hormone.

## 2. Materials and Methods

**Figure 1:**
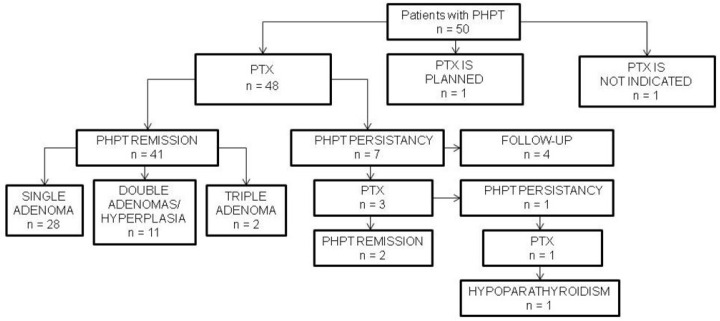
Patient’s flow chart. PHPT, primary hyperparathyroidism; PTX, parathyroidectomy.

This was a cohort study, which included patients with PHPT, who were referred to the Endocrinology Department of Almazov National Medical Research Centre from 2020 to 2023. PHPT was diagnosed according to current guidelines [21,22] based on biochemical criteria (elevated iPTH level and hypercalcemia OR twofold elevated iPTH level and normocalcemia; 25(OH)D > 30 ng/mL (>75 nmol/L); eGFR > 60 mL/min/1.73 m^2^); no patients with low 24 h urine calcium excretion (<200 mg/24 h) were included in the study. Genetic testing for MEN 1 syndrome was carried out in patients with clinical suspicious [23]. PNENs localization techniques were as follows: neck ultrasound, MIBI/[99mTc]-pertechnetate subtraction scintigraphy, CT, and PET/CT with [11C]-methionine or [11C]-choline. PTX was performed in 48 patients. The histologic examination of all removed PNENs confirmed them to be PAs. Among the operated-on patients, recurrent PHPT was observed in seven. In one patient, surgery was planned at the study completion time. In one case of ectopic paraesophageal PA, PTX was refused due to the high risk of esophagus damage; cinacalcet was prescribed with a positive effect. The patient’s flow chart is shown in Figure 1.

Intact PTH (iPTH, 15.0–65.0 pg/mL) assessment was carried out using immunochemiluminescent assay (Abbott Architect i2000SR, Chicago, IL, USA). The ionized and total calcium (1.11–1.32 and 2.15–2.65 mmol/L, respectively) were measured by autoanalyzer Abbot Architect c8000 (Chicago, IL, USA). Serum 25(OH)D level was detected by chemiluminescence immunoassay on micro particles (Abbott Architect c8000, Chicago, IL, USA). The 24 h urine calcium (2.50–8.00 mmol/24 h) was measured by the photometric absorption method in Cobas 6000 analyzer (Roche Diagnostics, Warsaw, Poland).

Neck ultrasonography, [99mTc]-MIBI/[99mTc]-pertechnetate subtraction scintigraphy, CT with contrast enhancement, and PET/CT with [11C]-methionine (or [11C]-choline) were performed according to previously described schemes [18,24].

[68Ga]-DOTA-peptides PET/CT was prospectively performed in patients with previously diagnosed PNENs, who signed an informed agreement for this examination. PET/CT was performed with the Scanner Discovery 710 (GE Healthcare, Milwaukee, WI, USA). Whole-body PET/CT acquisition was started 60 min after the intravenous injection of 350–600 MBq [68Ga]-DOTA-TATE or [68Ga]-DOTA-NOC. The choice of the tracer was determined by radiopharmaceutical availability at the time of the study. A parathyroid lesion was considered as a focus of high [68Ga]-DOTA-peptide uptake, which coincided with anatomical abnormalities according to CT-data.

Histological results served as the gold standard for labeling [68Ga]-DOTA-peptide findings as true or false positives. In cases where parathyroid lesions were not removed, coinciding with the [11C]-methionine PET/CT, CT, or scintigraphy imaging results with [68Ga]-DOTA-peptide positive findings were considered as true positives. This approach allows differentiating between parathyroid lesions and the stellate ganglia, both of which can accumulate [68Ga]-DOTA-peptide. However, the stellate ganglia do not accumulate MIBI or [11C]-methionine and exhibit different patterns of contrast media accumulation [25,26].

Moreover, the stellate ganglion has a precise localization. It lies in the neck between the longus colli muscle and the scalene muscles, positioned anterior to the transverse process and prevertebral fascia, trachea, esophagus, and vertebral column border it medially [27].

SST staining in formalin-fixed paraffin-embedded (FFPE) tissue blocks was carried out in 48 patients. Two of them were operated on before their admission to Almazov National Medical Research Centre and immunohistochemistry (IHC) was carried out in a retrospective manner. In the remaining 46 patients, PTX was performed in our centre. Commercially available rabbit monoclonal antibodies for SST2a (Clone UMB-1, Epitomics, Inc., Burlingame, CA, USA) and rabbit monoclonal antibodies for SST5 (Clone UMB-4 Epitomics, Inc., Burlingame, CA, USA) were used. A semi-quantitative method was used to assess SST expression. The expression of SST2 and SST5 on the tumor cell membrane and on the endothelium was investigated. The number of positive tumor cells was calculated and was scored as follows: “+++” (if >50%), “++” (if 30–50%), “+” (if 10–30%), “−” (if <10%). For the binary analysis, cases that scored ”−” or “+” were considered negative, while “++” and “+++” were considered positive for somatostatin receptor expression. The endothelial SST expression was classified as positive “+” or negative “−”.

STATISTICA 10.0 software (STATISTICA (RRID: SCR_014213) was used in statistical analysis). All variables demonstrated non-normal distribution; hence, non-parametric criteria were implemented. Continuous variables were characterized by medians (Me) and quartiles (25 to 75).

## 3. Results

### 3.1. Patients’ Clinical Characteristics

There were 50 patients with PHPT included in the study. The majority of patients were women (*n* = 43), F/M ratio was of 6:1, and median age was 64 (53;71) years. The patients’ laboratory and demographic characteristics are presented in Table 1. Characteristics of ten MEN1 patients are presented in Table A1. Genetic testing for MEN 1 syndrome was carried out in 32 patients. MEN1 gene variants were found in ten patients. Among the operated-on patients, recurrent PHPT was observed in seven and five of them had MEN1 syndrome. In patients with disease remission, histological examination confirmed single PAs in 24 cases and multiple PAs in 13 cases, and amongst all, 3 were atypical tumors.

### 3.2. [68Ga]-DOTA-Peptide PET/CT Results

Of the 16 patients who underwent [68Ga]-DOTA-peptides PET/CT, 11 were newly diagnosed and 5 had PHPT recurrence. Histology results were used as the gold standard to verify true positive findings in 12 cases where PET/CT with [68Ga]-DOTA-peptides was followed by PTX. In these patients, 21 PAs were removed. All 15 removed lesions with high [68Ga]-DOTA-peptide uptake were PAs. Six [68Ga]-DOTA-peptide PET/CT negative lesions appeared to be PAs. It is worth noting that four of them were not revealed by conventional imaging techniques and were only found through bilateral cervical exploration.

In 4 cases, PTX was not performed. In these cases, true positive PET/CT with [68Ga]-DOTA-peptides results were verified using CT (2 patients, 3 lesions), MIBI scintigraphy (1 patient, 1 lesion), and PET/CT with [11C]-methionine (1 patient, 2 lesions).

**Figure 2:**
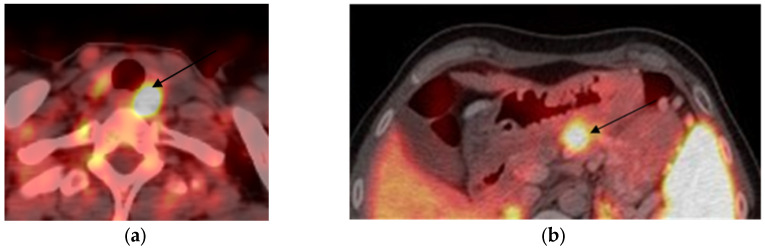
[68Ga]-DOTA-TATE PET/CT-scans: (**a**) [68Ga]-DOTA-TATE positive 11 × 7 × 13 mm lesion corresponded to left upper parathyroid adenoma (arrow); (**b**) [68Ga]-DOTA-TATE positive 37 × 30 × 36 mm lesion in pancreas head (arrow).

**Figure 3:**
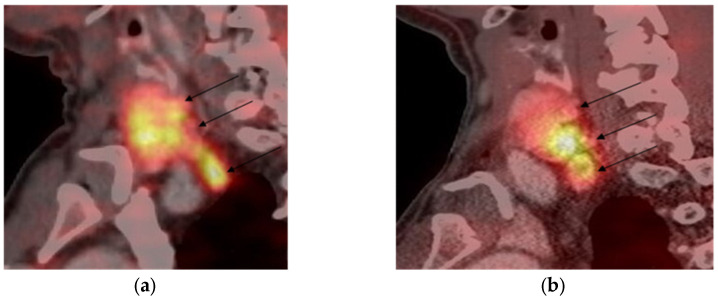
PET/CT-scans: (**a**) three [68Ga]-DOTA-TATE positive lesions in allocation of right upper (10 × 5 × 7 mm), middle (15 × 10 × 23mm), and lower (14 × 10 × 24 mm) parathyroid glands (arrows); (**b**) [11C]-choline PET/CT demonstrates all lesions have high [11C]-choline uptake (arrows).

PET/CT with [68Ga]-DOTA-peptide was performed in seven out of ten MEN1 patients (see patients one to seven in Table 2). Four patients already had PTX and were examined due to PHPT recurrence; three were newly diagnosed and underwent PET/CT with [68Ga]-DOTA-peptide before the initial surgery. Ten parathyroid lesions were found with conventional localization techniques, four via bilateral cervical exploration during PTX. Among all of them, seven were positive and seven were negative on PET/CT with [68Ga]-DOTA-peptide. Positive cases are shown in Figure 2 and Figure 3.

In total, 16 patients had 27 parathyroid lesions. In total, 20/27 lesions showed high [68Ga]-DOTA-peptide uptake. Seven PAs did not show [68Ga]-DOTA-peptide uptake, they were found by other imaging methods (three cases) or only during PTX (four cases); all of them were in MEN1 patients. The data are shown in Table 2.

### 3.3. SST2 and SST5 Staining Results

**Figure 4:**
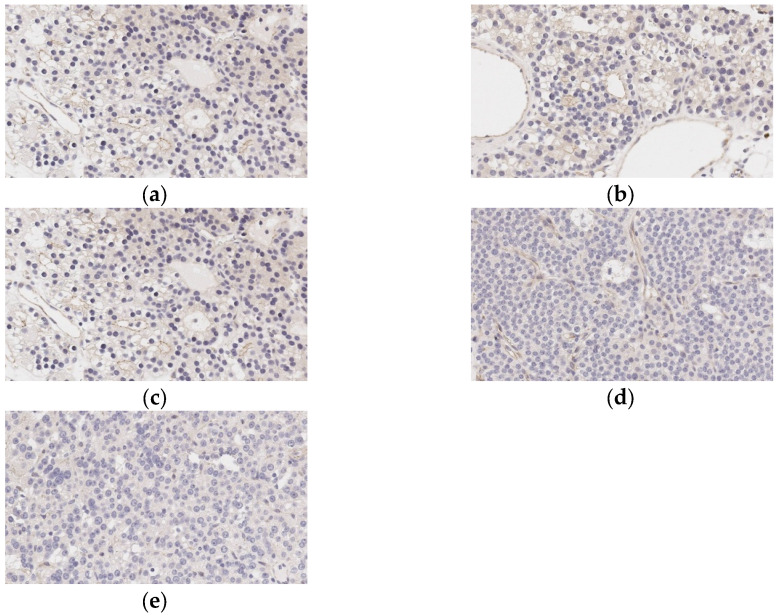
Immunohistochemical staining of SST5 in parathyroid neoplasms: (**a**) “+++” SST5-positive staining; (**b**) “++” SST5-positive staining; (**c**) “+” SST5-positive staining; (**d**) “−” SST5-negative staining; (**e**) SST5-positive staining in endothelium. For each panel: upper panel magnification, ×350; lower and middle panels’ magnification, ×400.

In 48 patients, PTX was performed. SST staining in FFPE tissue blocks was carried out in all cases (*n* = 48). None of them showed positive SST2 expression on parathyroid cell membranes. However, the majority expressed SST2 on endothelium (*n* = 39; 81%). SST5 staining was positive on both parathyroid cell membranes (*n* = 25; 52%) and endothelium (*n* = 40; 83%). The staining results are shown in Figure 4.

Twelve patients underwent PTX after [68Ga]-DOTA-peptides PET/CT scanning; in ten cases, [68Ga]-DOTA-TATE was used, and [68Ga]-DOTA-NOC was used in two. In eight cases, high [68Ga]-DOTA-TATE uptake was demonstrated by PAs, SST2 parathyroid cells membrane expression was not found in any case, while endothelial SST2 expression was observed in two adenomas. Both endothelium and parathyroid cells’ membranes SST5 expression were detected in all but two cases, in which only SST5 endothelial staining was observed. In two patients, PAs showed high [68Ga]-DOTA-NOC PET/CT uptake, but IHC revealed only endothelial SST2 and SST5 expression in both of them.

### 3.4. First-Generation Somatostatin Analogs’ Effects on Calcium and Parathyroid Hormone Values in PHPT Patients

**Figure 5:**
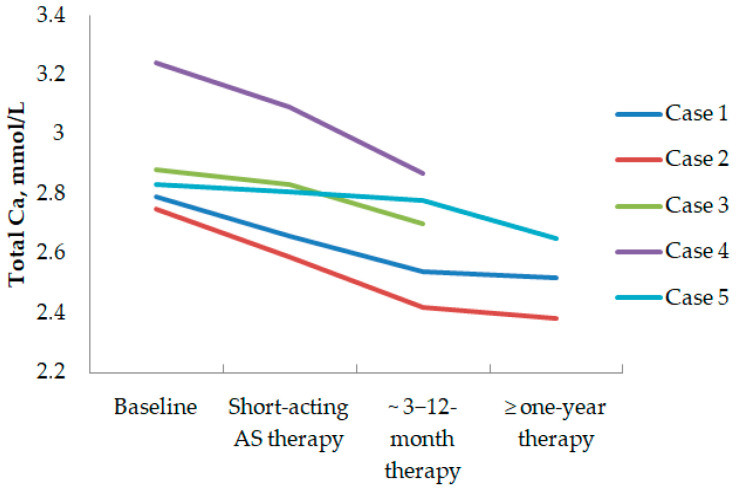
Total Ca level dynamics in patients treated with SSA1. Total Ca, total calcium; AS, somatostatin analogs.

**Figure 6:**
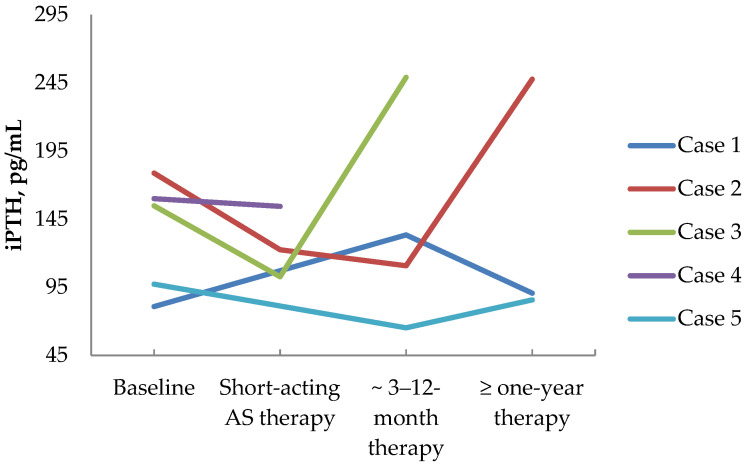
iPTH level dynamics in patients treated with SSA1.

In four MEN1 patients with persistent primary hyperparathyroidism after PTX, first-generation somatostatin analogs (SSA1) were prescribed for other neuroendocrine tumors. And in one patient with PHPT and growth hormone-secreting pituitary adenoma, SSA1 was prescribed due to persistent acromegaly. The patients’ data are shown in Table 3. Serum total Ca and iPTH levels influenced by SSA1 therapy are presented in Figure 5 and Figure 6, respectively. The following text provides detailed reports on all cases.

Case 1: In patient, 51 y.o., male, with MEN 1 syndrome (multiple duodenal and non-functioning pancreatic NETs, operated on chemodectoma, non-functioning adrenal adenoma, persistent PHPT after PTX—2 adenomas removed), long-acting SSA therapy was initiated (Lanreotide 120 mg/28 days) due to multiple pancreatic and duodenal NETs with uncertain malignant potential (with size up to 20 × 24 × 20 mm and Ki-67 2.6%). [68Ga]-DOTA-TATE PET/CT did reveal a PA with high radiopharmaceutical uptake. The long-term SSA1 effect on PHPT was assessed. In 8 mounts of Lanreotide use, the iPTH level raised a little: from 80.9 to 133.4 pg/mL (15.00–65.00). Meanwhile, in 1.5 years after SSA1 initiation, the iPTH level did not differ significantly from the baseline and was of 77.13 pg/mL (15.00–65.00). However, in 1.5 years, serum calcium slightly decreased: serum ionized Ca decreased from 1.51 to 1.34 mmol/L (1.11–1.29), serum total Ca decreased from 2.79 to 2.75 mmol/L (2.15–2.65), which corresponded to albumin-adjusted total Ca of 2.6 mmo/L. These values were relatively consistent after 3 years of therapy: iPTH 90.70 pg/mL (15.00–65.00), serum total Ca 2.52 mmol/L (2.15–2.65), serum ionized Ca 1.38 mmol/L (1.11–1.29), albumin-adjusted calcium 2.43 mmol/L, while 24 h calciura was 7.69 mmol/24 h (2.5–8.0) and 25(OH)D level was 29 ng/mL. Over three years of SSA1 therapy, parathyroid adenoma had grown from 5 × 4.5 × 4 mm to 6.5 × 7 × 21mm and PTX was performed.

Case 2: Patient №2, 41 y.o., female, with MEN 1 syndrome (prolactinoma, operated on insulinoma in 1991, multiple duodenal and non-functioning pancreatic NETs operated on in 2021, non-functioning adrenal adenoma, persistent PHPT after subtotal PTX). [68Ga]-DOTA-TATE PET/CT results are shown in Figure 2. Before subtotal pancreatectomy, 2/3 stomach resection, duodenectomy preoperative short-acting SSA therapy was initiated (Octreotide 200 mcg/3 times daily). In 19 days of therapy before the surgery, the calcium and iPTH levels were assessed; no significant changes were found: iPTH varied from 178.8 to 172 pg/mL (15.00–65.00), serum total Ca varied from 2.75 to 2.82 mmol/L (2.15–2.65), serum ionized Ca varied from 1.55 to 1.46 mmol/L (1.11–1.29), and albumin-adjusted calcium varied from 2.5 to 2.7 mmol/L. However, after the surgery, in terms of Octreotide’s prescription extension, a decrease in iPTH and calcium levels was witnessed: iPTH 122.5 pg/mL (15.00–65.00), serum total Ca 2.59 mmol/L (2.15–2.65), and albumin-adjusted calcium 2.6 mmol/L. After hospital discharge, Octreotide was replaced by Lanreotide (120 mcg/28 days), and in two years of therapy, the SSA1 effect on calcemia remained stable: serum total Ca 2.38 mmol/L (2.15–2.65), serum ionized Ca 1.25 mmol/L (1.11–1.29), and albumin-adjusted Ca 2.44 mmol/L. However, the iPTH level rose up to 247.7 pg/mL (15.0–68.3), whereas 25(OH)D was normal at 30.4 ng/mL and 24 h calciura was 4.21 mmol/24 h (2.5–8.0). A little parathyroid adenoma growth was observed in two years of therapy from 11 × 7 × 13 mm to 13 × 7 × 14 mm.

Case 3: In patient № 3, 45 y.o., female, with MEN 1 syndrome (prolactinoma, non-functioning pancreatic NET operated on in 2003 with local recurrence and metastasis in parapancreatic lymph node, persistent PHPT after subtotal PTX), due to uncertain malignant potential of pancreatic NETs, short-acting SSA therapy (Octreotide 300 mcg/2 times daily) was started in the hospital. Two parathyroid PAs were found and one of them demonstrated [68Ga]-DOTA-TATE uptake. In 2 weeks of therapy, a decrease in iPTH and calcium levels was found: iPTH decreased from 154.8 to 102.8 pg/mL (15.0–65.0). However, serum total Ca (2.88 to 2.83 mmol/L (2.15–2.65)), ionized Ca (1.45 to 1.52 (1.11–1.29) mmol/L), and albumin-adjusted calcium (2.9 to 2.7 mmol/L) levels did not change. After hospital discharge, the patient continued Lanreotide (120 mcg/28 days) and serum Ca and iPTH levels were assessed in 3 mounts: iPTH—249 pg/mL (13.7–98.2), serum total Ca—2.7 mmol/L (2.1–2.55), and ionized Ca—1.59 (1.18–1.32) mmol/L.

Case 4: Patient № 6, 53 y.o., male, with MEN 1 syndrome (duodenal gastrinoma, multiple non-functioning pancreatic and gastric NETs, non-functioning adrenal adenoma, PHPT). According to imaging, the patient had three PAs with sizes of 10 × 5 × 7 mm, 15 × 10 × 23 mm, and 14 × 10 × 24 mm; the first one had [68Ga]-DOTA-TATE uptake. PET/CT results are shown in Figure 4. Due to the unknown malignant potential of multiple pancreatic and gastric NETs, gastrin production short-acting SSA therapy (Octreotide 100 mcg/2 times daily) was initiated. After 2 weeks of therapy, no substantial decrease in iPTH and calcium levels was observed: iPTH—from 160 to 154.4 pg/mL (15.0–65) and serum total Ca—from 3.24 to 3.09 mmol/L (2.15–2.65). PTX was postponed since the patient had aortic insufficiency and aortic root aneurysm, which needed to be managed first. After hospital discharge, the patient was recommended to continue Lanreotide (120 mcg/28 days). In 4.5 mounts, serum Ca levels were assessed: total Ca—2.87 mmol/L (2.18–2.60), albumin-adjusted calcium—2.69 mmol/L, and serum gastrin decreased from 1280 to 446 pg/mL (13–115).

Case 5: Patient, 53 y.o., male, with growth hormone-secreting pituitary adenoma’s persisting after surgery in 2022, gastric adenocarcinoma after surgery and chemotherapy in 2022, non-functioning adrenal adenomas, who tested negative for MEN1 syndrome. Due to acromegaly persistence, Lanreotide therapy (120 mcg/28 days) was recommended, whereas PTX was postponed due to gastric adenocarcinoma treatment. In a year of SSA1 therapy, a decrease in the iPTH level from 97.35 to 85.79 pg/mL (15.00–65.00), the serum total Ca from 2.83 to 2.65 mmol/L (2.15–2.65), the serum ionized Ca from 1.45 to 1.42 mmol/L (1.11–1.29), and the albumin-adjusted calcium from 2.82 to 2.47 mmol/L was found. PTX was performed in 2023, which was followed by an adequate iPTH drop.

## 4. Discussion

The results of our study confirmed the initial hypothesis that [68Ga]-DOTA-peptide PET/CT allows the detection of PNENs. In our series, all PNENs that underwent 68Ga]-DOTA-peptide PET/CT were PAs. All of them showed high [68Ga]-DOTA-peptides uptake on total-body PET/CT scans, regardless of MEN1 mutation status. To our knowledge, [68Ga]-DOTA-peptides PET/CT utilities in parathyroid tumor imaging have not been studied before in patients without MEN syndromes. Moreover, the results of [68Ga]-DOTA-peptides PET/CT in MEN patients with PHPT confirmed the ability of PNENs to accumulate these radiopharmaceuticals [15,28,29]. Patil V.A. et al. in a study of 30 MEN1 patients with PHPT, demonstrated [68Ga]-DOTA-peptides PET/CT sensitivity of 24.6% in PAs localization [15]. Lastoria et al. found high [68Ga]-DOTA-TATE uptake in all parathyroid lesions in 5 out of 15 MEN1 patients [29]. In another study, [68Ga]-DOTA-NOC uptake was found in two out of six parathyroid lesions in patients with MEN1 and MEN2A [28]. For the first time, we showed that all PAs in non-MEN1 patients had high [68Ga]-DOTA-peptides uptake, while only 50% of parathyroid lesions accumulate radiopharmaceutical in MEN1 patients. The pitfalls of parathyroid lesions imaging in MEN1 syndrome could be caused by various factors. One of them may be the competitive accumulation of radiopharmaceutical lesions by the larger or more active NENs of other localizations. It is well known that a high uptake of [68Ga]-DOTA-peptide by bigger tumors can compete with smaller ones, such as PAs [4]. In our study, all four patients with negative [68Ga]-DOTA-peptide PAs had [68Ga]-DOTA-peptide-positive pancreatic NETs. Lastoria et al. reported similar data: among ten patients with [68Ga]-DOTA-TATE negative parathyroid lesions, seven patients had [68Ga]-DOTA-TATE positive pancreatic lesions [29]. Another cause of negative [68Ga]-DOTA-peptide uptake by PNENs might be competitive radiopharmaceutical accumulation by the dominant lesion in the case of multiple PAs, which is typical for MEN1 syndrome. In our series, in two cases of multiple PAs, one lesion showed high [68Ga]-DOTA-peptide uptake while the other did not. The evidence of the parathyroid NENs’ ability to accumulate [68Ga]-DOTA-peptides initiated the study of SST2 and SST5 expression in PAs.

In previous research, the expression of SSTs types 1–5 was investigated in PAs, atypical adenomas, and carcinomas. SST5 was postulated by the authors as a potential marker for malignancy [30]. According to our data, the cells of the PAs’ did not show membrane SST2 expression in any cases, including those that were [68Ga]-DOTA-TATE positive. Only endothelium SST2 expression was detected in the majority of stained PAs samples (39/48). SST5 membrane expression was positive in 52% and endothelial was positive in 83%. Thus, we did not confirm the role of SST5 in the prediction of parathyroid malignancy. Also, we found a discrepancy between the results of [68Ga]-DOTA-peptides PET/CT and IHC. In our eight patients with high [68Ga]-DOTA-TATE uptake, SST2-IHC expression on parathyroid cell membranes was negative, and SST5-IHC expression on parathyroid cell membranes was negative in two patients who had positive [68Ga]-DOTA-NOC PET/CT scans of PA. Despite the known fact that [68Ga]-DOTA-peptides uptake correlates with SSTs expression on neuroendocrine tumor cells’ membranes [31], the mismatch of results of functional (PET/CT) and IHC evaluation was observed before [32]. In the retrospective observational study, the correlation between the results of SSTs PET/CT and SST2 and SST5 IHC staining in the G1-G2 lung NETs was evaluated [32]. A correlation between SST2 IHC and PET/CT was found in 24/32 cases (75.0%, *p* = 0.003); in 8 cases, SST2 IHC was negative while [68Ga]-DOTA-peptides PET/CT was positive [32]. It should be noted that [68Ga]-DOTA-NOC and [68Ga]-DOTA-TOC were both used in PET/CT in the mentioned study [32]. According to our knowledge, a correlation between [68Ga]-DOTA-peptides PET/CT and SST2–IHC and SST5–IHC results has never been evaluated before in PAs. Our data suggests that in some NENs, high [68Ga]-DOTA-peptides uptake is not mediated by the tumor cells’ SST membrane expression, but probably by endothelial SST expression. These findings need further investigation.

Our data demonstrated the effectiveness of whole-body [68Ga]-DOTA-peptides PET/CT scanning in the PNENs imaging nevertheless it could hardly be recommended as a routine parathyroid lesion diagnostics method since it did not show higher sensitivity than conventional imaging techniques. However, regardless of the MEN syndrome’s positive state, lesions with high radiopharmaceutical uptake may be found in the parathyroid glands’ allocation during the whole-body [68Ga]-DOTA-peptides PET/CT scanning, which is performed for different reasons [14]. These focuses may be considered to be PNENs because it was shown before that normal parathyroid cells neither express SST2 and SST5 [33], nor demonstrate detectable [68Ga]-DOTA-TATE uptake on PET/CT scans [14]. Therefore, PHPT biochemical diagnostics may be initiated in such cases, and if the diagnosis is confirmed, we assume that no other functional imaging is needed. According to our data, not all lesions may accumulate radiopharmaceuticals in multiple PAs, namely in patients with MEN1 syndrome, so in such cases contrast-enhanced CT may be recommended for searching [68Ga]-DOTA-peptides negative parathyroid NENs. Such an approach is supported by the data of Patil et al. as they demonstrated that contrast-enhanced CT has higher sensitivity than [68Ga]-DOTA-NOC/TATE PET/CT while detecting parathyroid lesions in patients with MEN1 syndrome [15].

As we found high [68Ga]-DOTA-peptide uptake on PAs PET/CT scans, it was interesting to evaluate the effect of SSA1 on the calcium and parathyroid hormone levels. However, Octreotide and Lanreotide are not approved for PHPT treatment, so we could not prescribe them to patients with isolated PAs. Meanwhile, there were four MEN1 patients who received Lanreotide or Octreotide therapy due to gastrointestinal and/or pancreatic NETs and one acromegaly patient with excluded MEN1 syndrome by genetic testing, who was treated by Lanreotide due to pituitary disease persistence after transsphenoidal surgery. All these patients had PAs and PHPT as well, so we could evaluate calcium and parathyroid hormone changes during the first-generation SSA therapy. Despite the fact that a lowering of total serum calcium levels (see Figure 5) was observed, the parathyroid hormone level did not decrease, but even increased in most cases (see Figure 6). This may indicate that the decrease in calcium level did not occur through lower parathyroid hormone levels. Previous data on the impact of SSA on isolated PHPT is highly limited. We found only one study, published in 1995, that included 40 patients with PHPT [34]. Ocreotide (200 mcg) was applied intravenously once in all patients, and serum calcium, phosphate, and parathyroid hormone were assessed before the injection and four hours after. No significant changes in any of the investigated biochemical parameters were observed [34]. The results of this study were confirmed by our data to show that there is no inhibiting effect of SSA on parathyroid hormone production. However, the recently published research of Hansen et al. shows the possible underlying mechanisms of the opposite SSA effect on calcium and parathyroid hormone levels in patients with PHPT [35]. They showed that SST2 is expressed on human osteoclasts and itsactivation does not change osteoclast differentiation but decreases osteoclast activity [35]. Therefore, we may assume that the calcium-lowering effect of SSA therapy in patients with PHPT is the result of osteoclasts inhibition; in this case, the mechanisms of parathyroid hormone elevation level become clear. A decrease in serum calcium levels can lead to the activation of calcium-sensitive receptors located on the cells of the PAs and results in increased parathyroid hormone secretion. Consequently, PA growth may be stimulated in some cases, as we observed in Case 1 from our series. This should be taken into account when monitoring PHPT in patients receiving SSA therapy.

The major limitation of the study is a small sample size, which was determined by economic factors and patients’ unwillingness to undergo additional radiological procedures. Another factor that could affect the results is that in a few cases PET/CT scans were compared to other visualization results but not to pathology outcomes. Among PNENs in our cohort, there was no parathyroid carcinoma, so we could not obtain information on the [68Ga]-DOTA-peptides uptake and SST expression in parathyroid carcinoma. Further prospective studies are needed to establish a place for [68Ga]-DOTA-peptides’ PET/CT appliance in PNENs diagnostics.

## 5. Conclusions

The results of our study confirmed that PET/CT with [68Ga]-DOTA-peptides may detect both sporadic and MEN1-associated PNENs. Nevertheless, it could hardly be recommended as a routine method for PA imaging because it did not show better potential in PNEN imaging than conventional imaging techniques. Our data proves the hypothesis that incidentally localized lesions with high [68Ga]-DOTA-peptides uptake in the parathyroid glands’ allocation revealed during whole-body [68Ga]-DOTA-peptides PET/CT scanning, performed for different reasons, may be a reason for further biochemical evaluation for PHPT. If the latter is confirmed, additional parathyroid imaging is not necessary, and surgery may be considered. Since there is no correlation between PA high [68Ga]-DOTA-peptides uptake and expression of SST2 on parathyroid cells membranes, a new question arises: is this phenomenon a common feature of PNENs or can it also occur in other NENs? This needs further investigation. The effect of SSA therapy on calcium and parathyroid hormone levels as well as on the size of PAs in patients with MEN1 syndrome need further assessment.

## Figures and Tables

**Table 1:** General characteristics of patients.

Variables	Me (Min–Max)
age (years)	65 (21–87)
weight (kg)	74 (45–107)
height (cm)	162 (147–192)
BMI (kg/m^2^)	26 (16–40)
iPTH (pmol/L)	199 (73–1456)
serum iCa (mmol/L)	1.6 (1.3–2.1)
serum total Ca (mmol/L)	2.9 (2.5–3.6)
24 h calciura (mmol/24 h)	6.3 (4.3–24.1)
serum P (mmol/L)	0.8 (0.5–1.3)
25(OH)D (ng/mL)	28 (11–87)
eGFR ^1^ (mL/min/1.73 m^2^)	81 (47–123)

^1^ Calculated by CKD-EPI formula. BMI, body mass index; iCa, ionized calcium; total Ca, total calcium; 24 h calciura, 24 h urine calcium; P, phosphorus; iPTH, intact PTH; 25(OH)D, 25-hydroxyvitamin D; eGFR, estimated glomerular filtration rate.

**Table 2:** PET/CT with [68Ga]-DOTA-peptides’ results.

Patient № ^1^	Gender, Age	Histology Result	Size, mm	[68Ga]-DOTA- Peptides Uptake
1	M, 51 y.o.	adenoma	6.5 × 7 × 21	Neg.
2	F, 41 y.o.	NA	13 × 7 × 14	Pos.
3	F, 45 y.o.	NA	14 × 6.5 × 29	Pos.
			9.2 × 5.4 × 13.2	Neg.
4	F, 36 y.o.	adenoma	14 × 11 × 9	Pos.
		atypical tumor	7 × 6 × 12	Neg.
		adenoma	4 × 3 × 3	Neg.
5	F, 56 y.o.	adenoma	8.5 × 5.5 × 7	Pos.
6	M, 53 y.o.	adenoma	10 × 5 × 7	Pos.
		double adenoma	15 × 10 × 23	Pos.
		adenoma	14 × 10 × 24	Pos.
7	F, 21 y.o.	adenoma	8 × 4 × 13	Neg.
		adenoma	5 × 6 × 6	Neg.
		adenoma	6 × 7 × 8	Neg.
8	M, 53 y.o.	adenoma	15 × 10 × 18	Pos.
		adenoma	27 × 9 × 12	Pos.
9	F, 81 y.o.	adenoma	8 × 7 × 13	Pos.
10	M, 66 y.o.	adenoma	22 × 13 × 16	Pos.
		atypical tumor	44 × 48 × 54	Pos.
11	F, 81 y.o.	adenoma	13 × 12 × 8	Pos.
		adenoma	8.5 × 7 × 5	Pos.
12	F, 63 y.o.	NA	5.1 × 5 × 6.7	Pos.
		NA	5.9 × 3.7 × 5.3	Pos.
13	F, 54 y.o.	adenoma	18 × 22 × 27	Pos.
14	F, 65 y.o.	NA	20 × 11 × 44	Pos.
15	F, 71 y.o.	adenoma	18 × 10 × 22	Pos.
16	F, 71 y.o.	atypical tumor	27 × 26 × 41	Pos.

^1^ First 7 patients have multiple endocrine neoplasia syndrome type 1. NA, not applicable in patients since parathyroidectomy was not performed; y.o., years old; Neg., negative; Pos., positive; M, male; F, female.

**Table 3:** Clinical data of PHPT patients treated with first-generation somatostatin analogs.

Case №	Gender, Age	Indications for the Prescription of Somatostatin Analogs	Somatostatin Analogs’ Treatment Regimen	PHPT History
1	M, 51 y.o.	Multiple duodenal and non-functioning pancreatic NETs	Lanreotide 120 mg/28 days	Persistent PHPT after PTX (2 adenomas removed)
2	F, 41 y.o.	Multiple duodenal and non-functioning G2 pancreatic	Initially: Octreotide 200 mcg/3 times daily. Long-term: Lanreotide 120 mg/28 days	Persistent PHPT after subtotal PTX
3	F, 45 y.o.	Non-functioning pancreatic NET operated on in 2003 with local recurrence and metastasis in parapancreatic lymph node	Initially: Octreotide 300 mcg/2 times daily Long-term: Lanreotide 120 mg/28 days	Persistent PHPT after subtotal PTX
4	M, 53 y.o.	Duodenal gastrinoma, multiple non-functioning pancreatic and gastric NETs	Initially: Octreotide 100 mcg/2 times daily Long-term: Lanreotide 120 mg/28 days	PHPT before the surgery (3 adenomas)
5	M, 52 y.o.	Growth hormone-secreting pituitary adenoma’s persistence after surgery in 2022	Lanreotide 120 mg/28 days	PHPT before the surgery (2 adenomas)

Y.o., years old; M, male; F, female; PHPT, primary hyperparathyroidism; PTX, parathyroidectomy; NET, neuroendocrine tumor; case №, case number.

## Data Availability

The data presented in this study are available on request from the corresponding author due to privacy and ethical restrictions.

## Appendix A

**Table A1:** Clinical characteristics of patients with MEN 1 syndrome.

Patient № *	Gender, Age ^1^	PHPT Status	Other MEN1 Manifestations
1	M, 51 y.o.	remission after subtotal PTX	multiple duodenal and non-functioning pancreatic NETs, chemodectoma, non-functioning adrenal adenoma
2	F, 38 y.o.	recurrence after subtotal PTX	prolactinoma, insulinoma, multipal doudenal and non-functioning pancreatic NETs, non-functioning adrenal adenoma
3	F, 41 y.o.	recurrence after subtotal PTX	prolactinoma, multiple doudenal and non-functioning pancreatic NETs
4	F, 36 y.o.	remission after subtotal PTX	prolactinoma, multiple doudenal and non-functioning pancreatic NETs
5	F, 55 y.o.	hypoparathyroidism after subtotal PTX	non-functioning pituitary adenoma, multiple non-functioning pancreatic NETs
6	M, 53 y.o.	remission after subtotal PTX	duodenal gastrinoma, multiple non-functioning pancreatic, gastric NETs, non-functioning adrenal adenoma
7	F, 21 y.o.	remission after subtotal PTX	prolactinoma, multiple non-functioning pancreatic NETs
8	M, 31 y.o.	recurrence after 1 adenoma removal	prolactinoma, multiple non-functioning pancreatic insulinoma, non-functioning adrenal adenomas
9	M, 61 y.o.	recurrence after 1 adenoma removal	non-functioning pancreatic NETs, non-functioning adrenal adenomas
10	M, 66 y.o.	remission after subtotal PTX	prolactinoma, multiple non-functioning pancreatic NETs

^1^ Patient’s age at the time of genetic testing. * First 7 patient have multiple endocrine neoplasia syndrome type 1. y.o., years old; M, male; F, female; PHPT, primary hyperparathyroidism; PTX, parathyroidectomy; MEN1, multiple endocrine neoplasia syndrome type 1; NET, neuroendocrine tumor.
